# Inbreeding depression does not increase in foreign environments: a field experimental study

**DOI:** 10.1093/aobpla/plu009

**Published:** 2014-02-28

**Authors:** Joe Hereford

**Affiliations:** 1 Department of Biological Science, Florida State University, Tallahassee, FL 32306, USA; 2 Present address: Department of Evolution and Ecology, University of California, Davis, Davis, CA 95616, USA

**Keywords:** Dispersal, gene flow, inbreeding, inbreeding depression, local adaptation, reciprocal transplant.

## Abstract

Early successional species often disperse to novel environments, and if they are selfing, this dispersal will frequently be carried out by inbred individuals. If inbred immigrants are less likely to successfully establish populations than outbred immigrants, dispersal will be less effective and mating system evolution will favour outcrossing. I performed a reciprocal transplant of inbred and outbred plants grown in native and foreign planting sites to test the hypothesis that inbred immigrants had lower fitness. Inbreeding within populations was estimated with allozyme loci to confirm that the populations were inbred. While inbred and outbred plants had significantly lower fitness in foreign habitats, inbreeding depression was of similar magnitude at native sites and foreign habitats. There was no significant difference between inbred and outbred plants at foreign sites of the native habitat. Populations appear to be highly selfing, yet there is an advantage to outcrossing in both the native environment and foreign environments. The implications of this advantage with respect to mating system evolution may depend on whether novel environments are occupied or unoccupied.

## Abstract (precis)

Inbreeding depression can lower individual fitness and cause the extinction of populations. As a result, it is of interest to evolutionary biologists and conservationists alike. Studies have shown that inbreeding depression can increase in stressful environments. However, most of these studies do not utilize natural environmental stress. Hereford tested how natural environmental stress from transplanting into foreign habitats influences inbreeding depression. While there was significant inbreeding depression, there was no difference in inbreeding depression between plants in their native environment versus foreign habitats. These results imply that inbreeding depression does not increase when environmental stress reflects natural variation.

## Introduction

Inbreeding depression, the loss in fitness or performance associated with increased homozygosity, can shape the evolution of mating systems, dispersal strategies and absolute fitness. Given its importance, it is not surprising that there have been so many empirical estimates of inbreeding depression (Husband and Schemske 1996; Crnokrak and Roff 1999; Keller and Waller 2002; Scofield and Schultz 2006). Inbreeding depression is the major factor limiting the evolution of self-fertilization in plants (Jain 1976; Lande and Schemske 1985; Cheptou and Mathias 2001). In addition to avoidance of kin competition, avoidance of inbreeding depression in progeny fitness is a major factor in the evolution of dispersal ability (Levin *et al.* 2003; Roze and Rousset 2005; Guillaume and Perrin 2006). The mutation load that ultimately results in inbreeding depression can limit the absolute fitness of populations, increasing the probability of extinction (Ellstrand and Elam 1993; Mills and Smouse 1994; Frankham 2005).

One aspect of inbreeding depression that is not well understood is the role of inbreeding depression during immigration. Individuals colonizing new environments experience novel biotic and abiotic variables that could decrease fitness. The initial stages of colonization may be stressful for many populations because long-distance dispersal is often associated with lower fitness (Nathan 2006). Furthermore, many studies have reported lower fitness of migrants relative to natives (i.e. local adaptation; Linhart and Grant 1996; Hereford 2009*a*). When inbred individuals disperse into novel environments, they may face additional inbreeding depression because they are not genetically adapted to those environments, and inbreeding depression can increase under stressful conditions (Dudash 1990; reviewed in Armbruster and Reed 2005; but see Waller *et al.* 2008). Schmitt and Gamble (1990) showed in the annual plant *Impatiens capensis* that inbreeding depression increased with distance from the maternal parent at a local scale. Inbred progeny had significantly lower fitness than outcrossed plants 12 m from the seed parent. It may be more difficult for inbred individuals to establish in novel environments relative to outcrossed individuals. This ‘migration depression’ (Holsinger 1986) may be common in colonizing species that attempt to establish in new habitats. Along a spatial environmental gradient, inbreeding depression might increase with distance from a population's native site. If a population is locally adapted, then dispersal to a distant site would be more stressful than dispersal to a closer site. This could result in an increase in inbreeding depression in distant sites due to increased environmental stress.

Alternatively, inbreeding depression may not increase in distant or novel environments. First, novel environments may be so stressful that most individuals have low fitness (Angert and Schemske 2005). Here inbreeding depression is limited not from a lack of stress, but from a lack of opportunity for inbreeding depression and low overall variance in fitness (Waller *et al.* 2008). Another factor that might result in no increase in inbreeding depression in novel environments is if the novel environment is not stressful enough to increase inbreeding depression. Many studies have not detected an effect of environmental stress on inbreeding depression (Armbruster and Reed 2005). Furthermore, Fox and Reed (2011) showed that inbreeding depression increased linearly with the magnitude of environmental stress. Therefore, immigration into less stressful novel environments might induce less of an increase in inbreeding depression than immigration into more novel environments. Finally, some populations have greater relative and absolute fitness in non-native environments than in their local environment (Antonovics and Primack 1982; Galloway and Fenster 2000; Hereford and Winn 2008). This is especially common in host–parasite systems (Hoeksema and Forde 2008). Under these conditions, novel environments are not expected to be stressful and inbreeding depression should not increase.

I tested the hypothesis that inbreeding depression increases in novel environments. This study is not a direct test of the hypothesis that inbreeding depression increases with environmental stress (e.g. Dudash 1990), because foreign environments are not always more stressful. Instead, this is an experimental test of the interaction between inbreeding depression and dispersal in natural populations. Inbred and outcrossed offspring were generated by hand pollinations within each of six populations and then transplanted into the native sites of all six populations to compare the fitness of inbred and outbred progeny in novel environments. Inbreeding within populations was measured using allozyme loci to confirm morphological evidence that the study species is highly selfing (J. Hereford, pers. comm.) and that the populations are inbred.

## Methods

### Study system

*Diodia teres* is an early successional, self-compatible annual. The flowers are small (average <5-mm diameter), and individuals readily set seed in the greenhouse without the aid of pollinators. They are frequently visited by bees and Lepidopterans (J. Hereford, pers. comm.). Although there is no known method of long-distance dispersal in this species, it is widespread and appears to readily colonize suitable environments. Populations of this species have been shown to be locally adapted to their native habitats or planting sites (Jordan 1992; Hereford and Winn 2008) and to differ in the expression of maternal effects (Hereford and Moriuchi 2005). There are significant differences in allozyme allele frequencies among populations, suggesting limited gene flow (Hereford 2009*b*). The range of *D. teres* extends from Panama north to most of the central and eastern USA and into Arizona (Kearney and Peebles 1964). In the southeastern USA, populations occur in regions from the coastal plain to the Piedmont. The populations in this study have been described previously (Hereford and Winn 2008). They occur in three habitats: Dunes, Sandhills and Inland. Dunes have sandy soils and little canopy or herbaceous cover. Inland habitats have clay soils and more herbaceous and canopy cover. Sandhills are intermediate in soil texture, herbaceous cover and canopy cover. I studied two populations from each of these habitats. The populations are between 17 and 162 km apart. There is evidence that populations tend to be locally adapted to their native habitat type, but some populations had lower fitness in their native site than foreign populations (Hereford and Winn 2008).

### Measuring inbreeding

Allele frequencies at allozyme loci were measured to quantify inbreeding within populations. Methods of screening and sampling populations for allozyme variation have been previously described (Hereford 2009*b*). Four systems yielded six polymorphic loci. These systems were phosphoglucomutase (PGM), phosphoglucoisomerase 1 and 2 (PGI1, PGI2), glutamate dehydrogenase (GDH), and acid phosphatase 1 and 2 (ACP1, ACP2). Electrode buffer system 4 from Soltis *et al.* (1983) was used to resolve PGM and GDH, and a modification of the Soltis *et al.* (1983) system 6 was used to resolve PGI and ACP enzymes.

Between 14 and 17 individuals were sampled from each population. To estimate inbreeding, I calculated observed and expected heterozygosity, as well as Wright's measure of inbreeding within subpopulations, *F*_IS_, using the software package GDA (Lewis and Zaykin 1998). Deviation from random mating (possible inbreeding) is indicated by lack of fit to expectation of Hardy–Weinberg equilibrium. I tested for Hardy–Weinberg equilibrium using Fisher's exact test at each locus within each population. I calculated confidence intervals by bootstrapping with 1000 replicates over loci using the procedures and software in GDA (Lewis and Zaykin 1998).

### Inbreeding depression and local adaptation

In December of 2000, I collected seeds from each of the six populations to establish maternal families. I germinated these seeds in March of 2001. Plants from this generation were haphazardly assigned to act as pollen recipients (target plants) or pollen donors. To generate outcrossed seeds I hand pollinated a target plant with a pollen donor from the same population. Outcrossed hand pollinations were performed by emasculating flowers the day before anthesis and pollinating them the next morning (as in Hereford 2009*b*). I generated inbred progeny by allowing plants to self-fertilize without any manipulation. This was possible because emasculated flowers never set seed in the greenhouse unless they are hand pollinated. Therefore, I could be confident that the inbred crosses were truly self-fertilized. I collected selfed and outcrossed seeds in December of 2001, and then germinated those F1 seeds in March of 2002. I allowed both the selfed and the outcrossed F1 progeny to self-fertilize in the greenhouse so that I would have a large number of second-generation seeds of both types. Therefore, both selfed and outcrossed treatments experienced an additional generation of self-fertilization in the greenhouse before being planted in the field. The outcrossed treatment had a minimum inbreeding coefficient of 0.50. This step was necessary because *D. teres* produces only two seeds per flower and there would not have been the necessary replication within families in the F1 generation. The benefit of this step is that the outcrossing treatment more accurately mimics a natural outcrossing event. In a selfing species, few individuals are likely to be completely outcrossed, and any outcrossing is likely to be followed by additional selfing. This design is unusual in that the outcrossed plants are inbred, but they are less inbred than the selfed plants.

Methods of germination and planting have been described previously (Hereford and Winn 2008; Hereford 2009*b*). The F2 seeds were germinated in February of 2003 and the seedlings were grown in flats in the greenhouse. Seedling germination was carried out sequentially and all seeds from a maternal family were not germinated at the same time, as there was not enough space to germinate and maintain all seedlings at once. Germination occurred from February through March. After seedlings had produced at least two internodes, they were planted in the field. Planting date and initial size at planting were recorded and included as covariates in all statistical analyses. I planted members of all maternal families into all six planting sites from March through April. The seedlings were planted in randomized blocks at each planting site. Seedlings of the same maternal family and of the same type of cross were planted in haphazard order so that they were not all planted at the same time. When planting, I tried not to disturb natural vegetation, and there were no obvious differences in phenotype between the seedlings I planted and natural *D. teres* growing at all sites (J. Hereford, pers. comm.). On average, eight individuals from each inbred family and eight from each outcrossed family were planted at each field site. There was a total of 28 maternal families composed of inbred and outcrossed seedlings planted at all sites for a total of 2682 seedlings transplanted to the field. I planted seedlings from five families of Dunes population 1, seven from Dunes population 2, eight from Inland population 1, three from Inland population 2, three from Sandhills population 1 and two from Sandhills population 2. Therefore the sample size is large enough to detect inbreeding depression, but there is not enough replication to detect differences among populations. Finally, because all families were planted at all sites, variation among planting sites will not bias the results.

To estimate fitness I measured fruit production at the end of the growing season for each transplant. Plants that did not produce fruits were assigned a fitness of zero. Fruit production was measured in December of 2003, when more than 90 % of plants had senesced. Fruit production did not fit a normal distribution because of the inclusion of individuals that died before flowering. This resulted in a high frequency of zeros in the data, and the variance in fruit number was much greater than the mean. Therefore a generalized linear model assuming a negative binomial distribution was used to analyse effects on fruit production (McCullagh and Nelder 1989; cf. Hereford and Winn 2008).

Environmental differences are greater between planting sites of different habitats than between planting sites of the same habitat type (Hereford and Winn 2008). Given this variation, inbred progeny planted at foreign habitats should have lower fruit production than inbred progeny planted at the native site. Inbred plants at foreign habitats should also have lower fruit production than inbred plants at foreign sites of the native habitat. Finally, inbred plants should have lower fruit production than outbred progeny no matter where they are planted. I compared the fruit production of selfed and outcrossed plants grown in their native planting sites, foreign sites of the native habitat type, and foreign habitats. In the statistical model, the effects of initial size at planting, measured as total leaf area, and date of planting were accounted for. The effect of heterogeneity within planting sites was quantified by the block within planting site effect, and the effect of family was accounted for by the maternal family effect. Differences in fruit number produced by inbred and outbred progeny were quantified with the effect of cross type. The effect of planting treatment measures differences in fruit number resulting from being planted in alternate environments (native site, foreign site of the native habitat, and foreign habitat). The interaction between cross type and planting treatment measures how the effect of cross type depends on where the seedlings were planted. I investigated the nature of the interaction between cross type and planting treatment by making separate *post hoc* comparisons between inbred and outbred plants at native planting sites, foreign sites within native habitats, and foreign habitats. I used a version of the same statistical model described above, including planting date, planting site, block, maternal family and cross type but limited to each planting treatment. A significant effect of cross type with lower mean fruit number of inbred plants indicates significant inbreeding depression. I also compared the fruit number of inbred and outbred plants across planting treatments. This test was designed to test for differences in fruit number of inbred or outbred plants when planted at native sites, foreign sites of the native habitat type, and foreign habitats. These tests were similar to the above *post hoc* test of differences in fruit number between inbred and outbred plants at planting sites, but here there were paired comparisons between each type of planting treatment for inbred and outbred plants. A significant effect of planting treatment in this test indicates a difference in fruit number between a pair of planting treatments for a specific cross type.

## Results

### Inbreeding within populations

All loci indicate inbreeding given the low observed heterozygosity values (Table 1). The values of *F*_IS_ were all large and positive, indicating substantial inbreeding within populations. Only Inland population 1 was polymorphic at all loci. The other populations were segregating at two or three loci (Table 1). Observed heterozygosity was much lower than expected heterozygosity for all populations. All polymorphic loci in all populations significantly violated Hardy–Weinberg equilibrium except for GDH at Dunes population 2 and Sandhills population 2, and PGI1 and PGI2 at Inland population 2. The overall *F*_IS_ was 0.76 and was significantly different from zero with a confidence interval of (0.60, 0.96).

**Table 1.** Descriptive statistics of allozyme variation within populations. Observed heterozygosity (Obs. Het.), expected heterozygosity (Exp. Het.), inbreeding within populations (*F*_IS_) and number of loci polymorphic within each population (Loci) are displayed for each population. For all populations and loci, *F*_IS_ was significantly different from zero. Estimates per population are across all loci within each population, and estimates per locus are for each locus across all populations.

Population/locus	Obs. Het.	Exp. Het.	*F*_IS_	Loci
Inland 1	0.09	0.35	0.75	6
Inland 2	0.08	0.19	0.58	3
Sandhills 1	0.03	0.20	0.83	3
Sandhills 2	0.05	0.15	0.68	2
Dunes 1	0.02	0.14	0.93	3
Dunes 2	0.03	0.23	0.77	2
PGM	0.0	0.07	1.0	
PGI1	0.08	0.23	0.66	
PGI2	0.03	0.33	0.91	
GDH	0.21	0.42	0.52	
ACP1	0.00	0.08	1.00	
ACP2	0.00	0.11	1.00	
All populations	0.05	0.20	0.76	6

### Inbreeding depression and local adaptation

Earlier transplanting and larger initial size resulted in greater mean fruit number. Regression of date of planting on fruit number was negative (*β* = −0.20), and regression of initial size at planting was positive (*β* = 4.44). There were significant effects of maternal family and block within planting site on fruit number (Table 2). Removing the maternal family effect and replacing it with a maternal family nested within population effect and adding a source population effect did not change the qualitative outcome of any of the statistical models. Therefore, I did not include these effects in any of the analyses presented below.

**Table 2.** Generalized linear model of effects on fruit production. Planting treatment is divided into planting at the native site, foreign sites of the native habitat, and foreign habitat. The denominator degrees of freedom for all effects is 2591.

Source of variation	DF	χ^2^	*P* value
Initial size at planting	1	170.91	<0.0001
Planting date	1	22.71	<0.0001
Block within planting site	44	441.40	<0.0001
Maternal family	27	231.84	<0.0001
Planting site	5	645.81	<0.0001
Planting treatment	2	109.16	<0.0001
Cross type	1	1.88	0.1706
Planting treatment × cross type	2	10.36	0.0056

The overall mean fruit number of inbred progeny (14.10 ± 0.80 SE) was lower than the mean for outcrossed progeny (17.97 ± 0.84 SE). The mean fruit number of plants at their native sites was greater than when they were planted in all foreign sites, including foreign sites of the native habitat (18.59 ± 1.16 SE and 15.84 ± 0.67 SE, respectively).

**Figure 1.**
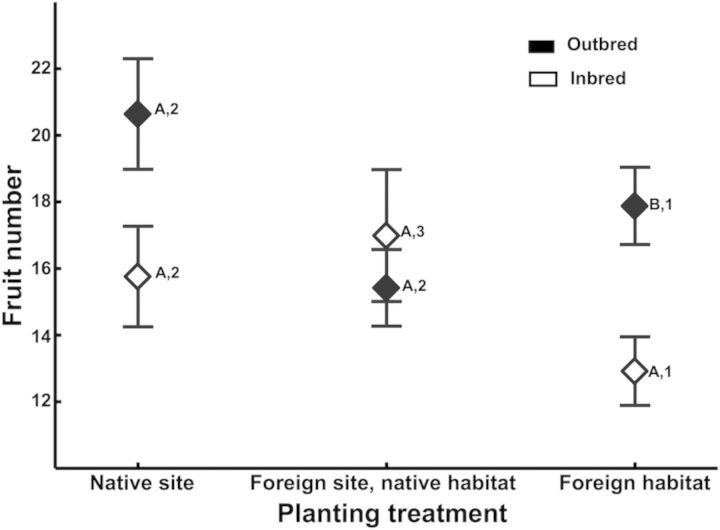
Mean fruit production of inbred and outbred progeny (averaged over families) planted at native planting sites, foreign sites of the native habitat type, and foreign habitat types. Scale bars indicate one standard error. Letters describe results of comparisons between means of inbred and outbred plants at each planting treatment, and numbers describe results of comparisons of within inbred and outbred plants across planting treatments. Means that share the same letter within a planting treatment and means that share the same number within a type of cross are not significantly different.

The difference between inbred and outbred fruit number depended on where they were planted, and there was a significant cross type by planting treatment interaction (Table 2). The difference in fruit number between inbred and outbred plants was not significant at native sites, but it was significant at foreign habitats (Fig. 1). I did not detect a significant difference in fruit number between inbred and outbred plants at foreign sites of the native habitat (Fig. 1). There was no increase in overall inbreeding depression at foreign habitats, as the difference in mean fruit production between inbred and outbred plants did not increase in foreign sites (Fig. 1). For inbred progeny, fruit production was significantly lower in foreign habitats than in native sites (significant effect of planting treatment, *P* = 0.0023), but greater in foreign sites of the native habitat than native sites (significant effect of planting treatment, *P* < 0.0001). For outbred progeny, fruit production was significantly lower at foreign habitats than native sites (significant effect of planting treatment, *P* < 0.0001). There was no significant difference in fruit production between native sites and foreign sites of the native habitat type for outbred progeny (non-significant effect of planting treatment, *P* = 0.3530).

## Discussion

Despite evidence of frequent inbreeding within populations (Table 1), inbred progeny had lower fruit production at native sites and foreign habitats than outbred progeny. I detected significant inbreeding depression only at foreign habitats, but its magnitude was similar to inbreeding depression at native sites (Fig. 1), and similar to inbreeding depression in other selfing angiosperms (Husband and Schemske 1996; Winn *et al.* 2011). Therefore, inbreeding depression did not increase from native to foreign sites. Lack of significant inbreeding depression at native sites may result from low statistical power. The magnitude of the difference between inbred and outbred plants in mean fruit production was similar at native sites and foreign habitats (4.89 versus 4.96 fruits), but the sample size of the native site comparison was much lower than all the other comparisons (196 versus 754 or more inbred progeny in all the other comparisons). Therefore, there was less statistical power to detect a difference of this magnitude. Inbreeding depression did not consistently increase with environmental difference. There was less inbreeding depression in foreign sites of the native habitat than there was at native sites (Fig. 1). The lack of inbreeding depression at foreign sites of the native habitat is surprising. It obviously does not result from a lack of phenotypic variance (e.g. Waller *et al.* 2008), but from similar fruit production in inbred and outcrossed progeny. Other studies of these populations show similar patterns, where fitness increases in foreign sites of the native habitat type (Hereford and Winn 2008; Hereford 2009*b*). Several mechanisms might generate this pattern, including variation in herbivory, but there is no evidence of differences in herbivory in inbred and outbred plants at any planting site (J. Hereford, unpubl. res.).

The genetic mechanism driving inbreeding depression in this species appears to be the accumulation of deleterious mutations within populations (e.g. Lande and Schemske 1985). There is significant and substantial genetic population structure between populations of *D. teres* (Hereford 2009*b*), and populations are inbred (Table 1). Crosses between the populations in this study reveal strong heterosis even in populations from different habitats (Hereford 2009*b*), and many populations are not locally adapted to their native habitat (Hereford and Winn 2008). Thus, populations retain a mutation load of deleterious alleles, but because there is likely to be little gene flow between populations, there is no mechanism to purge the deleterious mutations, and they can eventually fix within populations (Lynch *et al.* 1995). While this basis of inbreeding depression is most probable, other mechanisms such as overdominance, or inbreeding depression as a result of stabilizing selection in maladapted populations (Ronce *et al.* 2009) are also possible. Regardless of the dominant mechanism, it is also possible that multiple mechanisms of inbreeding depression can occur within a population.

Armbruster and Reed (2005) and Fox and Reed (2011) showed that inbreeding depression often increased with environmental stress. Fox and Reed (2011) concluded that inbreeding depression should increase linearly with environmental stress, and that studies that do not detect an increase in inbreeding depression with environmental stress may lack statistical power or that the environmental stress may be too weak to detect a difference. They concluded that studies with less than a 25 % decrease in fitness due to stress would not detect an effect of stress on inbreeding depression. The environmental stress of dispersing to a foreign site resulted in a 15 % reduction in fitness in this study, but unlike most of the studies in both reviews, the environmental stress was based on natural environmental variation. Given that the environmental stress has to be of such a strong magnitude to detect an increase in inbreeding depression, natural populations of *D. teres* may not frequently experience an increase in inbreeding depression with environmental stress. It is possible that inbreeding depression would have been greater in foreign habitats had the outcross progeny had a near-zero inbreeding coefficient. However, the overall magnitude of inbreeding depression was similar at native sites and foreign habitats.

Inbreeding depression may vary among years, and total inbreeding depression may have been underestimated. Inbreeding depression was measured for a single year. The estimates of fitness in inbred and outcrossed progeny may vary from year to year (Hayes *et al.* 2005), but there is no reason to suspect that some years could favour inbred plants and other years would favour outbred plants. In this study, the male component of fitness was not measured. Inbreeding depression has been shown to affect pollen production and siring success (e.g. Carr and Dudash 1997; Rausher and Chang 1999). Given that the populations in this study are highly inbred (Table 1) and most likely highly selfing, male fitness will have less of an effect on total inbreeding depression than if the populations were outcrossing. Therefore it is unlikely that total inbreeding depression was underestimated.

### Implications for metapopulation dynamics and mating system evolution

Populations of *D. teres* along with other selfing species may be subject to frequent extinction and recolonization as a result of the difficulty of selfers to colonize and establish in novel environments. The populations in this study may not be part of a classic metapopulation (Harrison 1991). However, colonization of new sites in this species involves dispersal and sometimes adaptation to new environments, which are recognized as factors that limit successful colonization (Sakai *et al.* 2001). Laboratory studies suggest that environmental stress increases the probability of extinction in small, inbred populations relative to outbred, non-stressed populations (Bijlsma *et al.* 2000; Reed *et al.* 2002). In the present study, inbred plants had lower fitness than outbred plants in foreign habitats, but there was no difference between them at foreign sites of the native habitat (Fig. 1). More dispersal will occur into foreign sites of the native habitat than to foreign habitats. Therefore, inbred plants will not be at a disadvantage when dispersing into foreign sites of the native habitat, but when colonizing a novel habitat outcrossing will be advantageous.

Theoretical models show that the probability of successful colonization of inbred and outbred immigrants can influence the direction of mating system evolution. Self-fertilization is favoured because it provides reproductive assurance when there are Allee effects (Baker 1955). In general, the advantages of selfing are diminished by inbreeding depression (Jain 1976; Lande and Schemske 1985; Charlesworth and Charlesworth 1987), but accounting for inbreeding depression of immigrants or in a metapopulation context can lead to counterintuitive theoretical results. In a model of mating system evolution in a metapopulation, Pannell and Barrett (2001) showed that increased cycles of colonization and extinction favoured outcrossers over selfers when selfers suffered inbreeding depression. Holsinger (1986) showed that if the fitness of inbred immigrants into occupied habitats was low relative to outbred progeny, mixed mating was stable, but the decrease in density associated with inbreeding depression in a metapopulation can lead to increased selection for selfing (Dornier *et al.* 2008). Reproductive assurance in *D. teres* is nearly complete (J. Hereford, pers. comm.). Nearly all unmolested flowers produce seeds. Flowers open in the morning with little to no self-pollen on the stigmas. In the late afternoon the flowers wilt and collapse covering the stigma with self-pollen. Outcrossing can occur if flowers are pollinated before they wilt. There is clearly a reproductive assurance advantage to selfing in this species when colonizing a new unoccupied site.

When colonizing an occupied site, there is an even greater advantage to outcrossing than the inbreeding depression quantified in this study. Any outcrossing into occupied foreign sites of the native habitat or in foreign habitats will result in much greater fitness than inbreeding or outcrossing within the same population. Interpopulation crosses planted at the same field sites produced almost twice as many fruits as the inbred crosses in this study (Hereford 2009*b*). This result suggests that if inbred immigrants that arrive into new sites are able to outcross with native individuals in an occupied site, they will enjoy an almost two-fold advantage in fruit production over selfing. This advantage could counteract the automatic transmission advantage of self-fertilization. Outcrossing is especially favoured if the new site is a foreign habitat because selfing would incur inbreeding depression and outcrossing with natives could result in heterosis (Hereford 2009*b*; Fig. 1). There is little evidence for an optimal outcrossing distance (e.g. Waser and Price 1994) in *D. teres*, as outbreeding depression has been detected in only a few crosses (Hereford 2009*b*). Yet populations are primarily inbred (Table 1), suggesting that the reproductive assurance advantage outweighs any dispersal-dependent benefits of outcrossing. Given the evidence of inbreeding (Table 1), and the evidence of infrequent gene flow (Hereford 2009*b*), there is little evidence that mating system evolution depends on dispersal and gene flow in *D. teres*. In species with more frequent gene flow, the extent to which mating system evolution depends on local adaptation may depend on rates of dispersal into novel environments and the fitness consequences of selfing versus outcrossing within those environments. Models of mating system evolution in metapopulations typically do not incorporate spatial environmental heterogeneity. Adding this component will lead to more complicated models, but may shed light on how adaptation in a metapopulation can influence mating system evolution.

## Sources of Funding

This work was supported by National Science Foundation award DEB 9903878 to A.A. Winn and National Science Foundation award DDIG 0407968 to J.H.

## Conflicts of Interest Statement

None declared.
